# Nutritional Programming in the Rat Is Linked to Long-Lasting Changes in Nutrient Sensing and Energy Homeostasis in the Hypothalamus

**DOI:** 10.1371/journal.pone.0013537

**Published:** 2010-10-21

**Authors:** Ricardo Orozco-Solís, Rhowena J. B. Matos, Omar Guzmán-Quevedo, Sandra Lopes de Souza, Audrey Bihouée, Rémi Houlgatte, Raul Manhães de Castro, Francisco Bolaños-Jiménez

**Affiliations:** 1 INRA, UMR1280 Physiologie des Adaptations Nutritionnelles, Université de Nantes, Nantes Atlantique Université, Nantes, France; 2 Departamento de Anatomia, Centro de Ciências Biológicas, Universidade Federal de Pernambuco, Recife, Pernambuco, Brazil; 3 INSERM, U915, IFR26, l'institut du Thorax, Nantes, France; 4 Departamento de Nutriçao, Centro de Ciências da Saude, Universidade Federal de Pernambuco, Recife, Pernambuco, Brazil; University of Córdoba, Spain

## Abstract

**Background:**

Nutrient deficiency during perinatal development is associated with an increased risk to develop obesity, diabetes and hypertension in the adulthood. However, the molecular mechanisms underlying the developmental programming of the metabolic syndrome remain largely unknown.

**Methodology/Principal Findings:**

Given the essential role of the hypothalamus in the integration of nutritional, endocrine and neuronal cues, here we have analyzed the profile of the hypothalamus transcriptome in 180 days-old rats born to dams fed either a control (200 g/kg) or a low-protein (80 g/kg) diet through pregnancy and lactation. From a total of 26 209 examined genes, 688 were up-regulated and 309 down-regulated (P<0.003) by early protein restriction. Further bioinformatic analysis of the data revealed that perinatal protein restriction permanently alters the expression of two gene clusters regulating common cellular processes. The first one includes several gate keeper genes regulating insulin signaling and nutrient sensing. The second cluster encompasses a functional network of nuclear receptors and co-regulators of transcription involved in the detection and use of lipid nutrients as fuel which, in addition, link temporal and nutritional cues to metabolism through their tight interaction with the circadian clock.

**Conclusions/Significance:**

Collectively, these results indicate that the programming of the hypothalamic circuits regulating energy homeostasis is a key step in the development of obesity associated with malnutrition in early life and provide a valuable resource for further investigating the role of the hypothalamus in the programming of the metabolic syndrome.

## Introduction

In the past two decades, there has been an important increase in the incidence of the metabolic syndrome worldwide. The constellation of metabolic abnormalities characteristic of this pathological entity includes abdominal obesity, glucose intolerance, dyslipidaemia, hypertension and artherosclerosis. The etiology of the obesity which is at the heart of the metabolic syndrome may not simply be a consequence of an imbalanced diet or of a sedentary lifestyle. Obesity is a multifactorial condition in which environmental, biological and genetic factors all have a contributory role [1]. Actually, infants born small for gestational age, as a result of a deficient provision of macro- and micronutrients during development, are at increased risk of developing obesity, insulin resistance, cardiovascular diseases and hypertension during adulthood [2]. To explain these observations, it has been hypothesised that the nutritional environment during the critical period of perinatal development programs whole body energy homeostasis for optimal survival under nutritionally-deficient conditions. However, if these early-life adaptations mismatch with the environmental conditions the individual will confront later in life they will favour metabolic disorders [3], [4]. In support of this hypothesis, experimental studies in several animal species have shown that the offspring of dams exposed to protein or global calorie restriction during pregnancy and/or lactation exhibit several physiological disturbances linked to the metabolic syndrome such as insulin resistance [5], [6], reduced leptin sensitivity [7], [8], hepatic steatosis [9], elevated blood pressure [10] and hyperlipidemia [5], [8], [9].

The actual mechanisms responsible for the perinatal programming of body weight homeostasis that then lead to obesity and altered metabolism at the whole body level are not well understood. Nevertheless, several lines of evidence indicate that perturbations in the control of appetite and energy homeostasis within the brain are involved. Notably, exposure to nutrient restriction during perinatal development results in offspring which are hyperphagic and exhibit enhanced preference for high-fat food [11], [12], [13], [14]. Meal pattern analysis has further indicated that the increased food intake presented by metabolic programmed rats is due to a delay appearance of satiety, an increase in meal size and a reduced latency to eat [15]. The offspring born to nutrient restricted dams exhibit also alterations in the hypothalamic expression levels of several neuropeptides regulating food intake [16], [17], [18] as well as reduced numbers of galanin and NPY neurons [19], [20]. The anorexic actions of insulin [21], leptin [12] and serotonin [12], [14] have also been shown to be reduced in the rat offspring of dams submitted to protein or calorie restriction. Taken together, these observations indicate that a dysfunction of the hypothalamic circuits regulating energy homeostasis and food intake plays a role in the developmental programming of the metabolic syndrome.

To get an integrated view of the molecular pathways that might underlie the behavioural and physiological perturbations of feeding behavior induced by perinatal undernutrition, here we have used DNA chips and quantitative RT-PCR to analyze the perturbations of the hypothalamic transcriptome induced by the exposure to a low protein diet through pregnancy and lactation. The analysis by gene ontology of the data revealed an over-representation of genes involved in cell signal transduction and the regulation of metabolic process and gene transcription.

## Results and Discussion

### Phenotypic characteristics of LP rats

At birth, the offspring born to dams fed the low protein diet weighed significantly less than their control counterparts (5.97±0.09 g vs 7.00±0.18; P<0.0001) but they showed later a delayed catch-up growth such that at the completion of the study no statistically significant differences in body weight were observed between the two groups (Table 1). In contrast, adult LP exhibited higher serum levels of cholesterol, triglycerides and free fatty acids along with increased abdominal fat and enhanced expression levels in liver of Fatty acid Synthase, Stearoyl-Coenzyme A desaturase 1 and Carbohydrate response element binding protein (Table 1). Collectively, these observations sustain the results of previous studies indicating that protein restriction during early life induces metabolic and body composition disturbances in adulthood [5], [6], [8], [9].

**Table 1:** Phenotypic characteristics of control and LP rats.

	Controls	Low protein	Statistical significance
**Morphological traits**			
Body weight (g)	682±33	646±24	NS
Abdominal fat (% of body weight)	4.64±0.30	6.91±0.44	P<0.01
**Serum metabolites**			
Cholesterol (mmol/l)	2.78±0.13	3.87±0.26	P<0.01
Triglycerides (mmol/l)	2.52±0.33	3.81±0.18	P<0.05
Fatty acids (mmol/l)	0.32±0.03	0.51±0.05	P<0.05
Glucose (mmol/l)	9.53±0.47	10.01±0.31	NS
Insulin (ng/ml)	7.31±1.50	3.91±0.82	NS
**Gene expression in liver**			
Fatty acid synthase (FAS)	0.88±0.08	1.50±0.09	P<0.05
Stearoyl-Coenzyme A desaturase 1 (SCD 1)	0.92±0.08	1.41±0.09	P<0.01
Carbohydrate response element binding protein (ChREBP)	1.02±0.07	1.26±0.04	P<0.05

### Gene ontology analysis of the global expression profile

Comparison of the gene expression profile of the 26 209 genes constituting the microarray, showed that a total of 997 genes were differentially expressed in the hypothalamus of LP rats versus that of control animals (P<0.003). Among them, 688 were up-regulated and 309 were down-regulated representing 2.6% and 1.2%, respectively, of the total number of analyzed genes (Figure S1). The functional classification of these genes using the DAVID data base further revealed that most of them belong to Gene Ontology categories corresponding to signal transduction, regulation of metabolic processes and gene transcription (Table 2). Interestingly, some of the functional categories were overrepresented (EASE score P<0.05), only in the up-regulated or in the down-regulated group. Thus, the functional categories associated with cellular signaling pathways (synaptic transmission, kinase activity, intracellular signaling cascade, steroid hormone receptor activity), and transcription processes (transcription, transcription DNA dependent, transcription regulatory activity, cell cycle process) were over-represented in the up-regulated group, whereas the functional categories related to metabolism (metabolic process, protein metabolic process, cellular homeostasis, oxidoreductase activity) and cell cycle were over-represented in the down regulated group. This observation, together with the fact that only ∼5% of the altered genes were classified into a unique functional category, suggests that perinatal protein restriction affects an array of genes that act as a network to control metabolism and other cellular processes rather than a series of independent signaling pathways.

**Table 2:** Functional gene ontology categories enriched in the hypothalamus of 180 days-old LP rats.

Gene ontology category	GO Number		UP-Regulated			Down-Regulated	
		Number	%	P-value	Number	%	P-value
Signal transduction	GO:0007165	109	16	0,4828	29	9	1,0000
Intracellular signaling cascade	GO:0007242	51	7	0,0041	0	0	-----
Steroid hormone receptor activity	GO:0003707	10	1	<0.0001	0	0	-----
Transcription	GO:0006350	86	13	<0.0001	26	8	0,5403
Transcription, DNA-dependent	GO:0006351	77	11	<0.0001	21	7	0,7604
Transcription regulator activity	GO:0030528	63	9	<0.0001	16	5	0,7708
Metabolic process	GO:0008152	206	30	0,0429	128	41	0,0030
Regulation of metabolic process	GO:0019222	85	12	<0.0001	32	10	0,3318
Protein metabolic process	GO:0019538	86	13	0,4753	61	20	0,0285
Cellular homeostasis	GO:0019725	8	1	0,7503	14	5	0,0012
Oxidoreductase activity	GO:0016491	0	0	-----	22	7	0,0149
Cell cycle process	GO:0022402	15	2	0,3093	15	5	0,0084
Synaptic transmission	GO:0007268	30	4	<0.0001	0	0	-----
Ion transmembrane transporter activity	GO:0015075	30	4	0,0247	8	3	0,8862
Neurogenesis	GO:0022008	20	3	0,0073	0	0	-----
Intracellular part	GO:0044424	199	29	0,0004	127	41	0,0005
Voltage-gated channel activity	GO:0022832	13	2	0,0031	0	0	-----
Ion binding	GO:0043167	126	18	<0.0001	35	11	0,8940
Kinase activity	GO:0016301	45	7	0,0001	8	3	0,9671

The number and percentage of genes up-regulated or down regulated within each GO category in LP rats in relation to control animals are presented. The indicated P-value corresponds to modified Fisher Exact P-Value for gene-enrichment analysis (EASE score). From a total of 26609 examined genes, 688 were up-regulated and 309 down-regulated.

**Figure 1:**
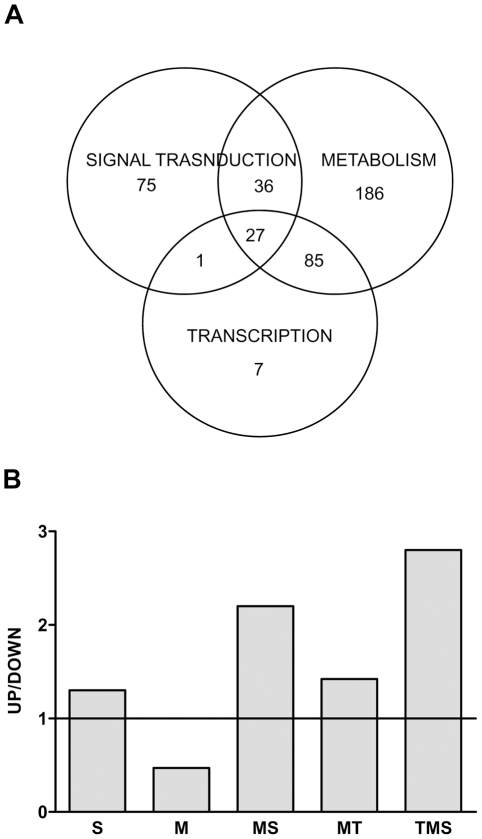
Functional classification (A) and global tendency of expression (B) of the genes differentially expressed in LP versus control rats. (A) Venn diagram showing the number of genes classified into one single GO category as well as the number of genes exerting two or more biological functions. (B) A big proportion of the genes whose mRNA expression levels are affected by protein restriction during early life possess more than one function and exhibit a tendency for over-expression. S =  signal transduction; M =  metabolic process; T =  transcription.

To further explore this hypothesis, we selected the most representative GO categories (signal transduction, S; transcription, T; and metabolic process, M), and looked for the genes classified into at least two of the selected GO categories (see methods). We reasoned that genes exerting two or more of these biological functions play an important role in the establishment and functioning of metabolic networks [22]. We found that 148 genes were classified into two or more GO categories (Figure 1A). Among them, 36 genes were classified into the signal transduction and metabolic process categories (SM), 85 into the transcription and metabolic process (MT), and 27 genes were classified into the three categories (TMS, Figure 1A). Some of the genes belonging to these overlapped areas are listed in Tables 3 and 4. All of them encode transcription factors, nuclear receptors or enzymes involved in the regulation of metabolism. To determine the global tendency of expression of the genes within each GO category, we calculated the ratio between the number of up- versus down-regulated genes in each category and their overlapped areas (Figure 1B). Interestingly, we found a ratio >1, indicating a tendency for over-expression, of the genes involved in the regulation of cellular signaling processes (S,1.3; MS,2.2; MT, 1.42. TMS, 2.8), and a ratio <1, indicating an inhibition of expression, of the genes involved in the regulation of metabolism (M, 0.47).This observation suggests the existence of “functional units” or “modules” [23] that are differentially altered by metabolic programming.

**Table 3:** Overview of gene expression changes in the hypothalamus of LP rats in relation to control animals.

Gene name	Gene ID	Fold change	P-value
**Metabolism**			
60S ribosomal protein L13	500276	−1,62	0,0009
Ribosomal protein L15	364239	−1,54	0,0024
Eukaryotic translation initiation factor 3	293484	−1,51	0,0008
Seryl-aminoacyl-tRNA synthetase 1	266975	−1,33	0,0018
Aspartyl-tRNA synthetase 2	304919	−1,23	0,0024
Lactate dehydrogenase 3	29634	2,30	0,0017
UDP-glucose pyrophosphorylase 2	289827	−1,19	0,0015
Alpha-mannosidase II	25478	2,64	0,0003
Malate dehydrogenase	81829	−1,34	0,0003
Xylosyltransferase 1.	64133	3,19	0,0005
Glucosidase 1	78947	−1,58	0,0017
Glutamine fructose-6-phosphate transaminase 1	297417	3,46	0,0002
Glutamate decarboxylase 2	24380	2,80	0,0009
N-acetylglucosaminyltransferase	65271	3,87	0,0006
Peptidylprolyl isomerase B	64367	−1,38	0,0019
Serine palmitoyltransferase	296188	2,48	0,0004
Cytochrome P450, family 3	171352	−1,77	0,0009
Cytochrome b-5	64001	−1,47	0,0010
Cytochrome P450, family 20	316435	−1,41	0,0015
Cytochrome oxidase deficient homolog 1	497930	−1,54	0,0025
Cytochrome c oxidase polypeptide VIII-liver.	680156	−2,56	0,0027
Cytochrome c oxidase subunit IV isoform 1	29445	−1,28	0,0028
Peroxiredoxin 1	683813	−2,31	0,0018
NADH dehydrogenase Fe-S protein 6	29478	−1,32	0,0022
**Signal transduction**			
Galanin receptor 1	50577	2,13	0,0002
Cannabinoid receptor 1	25248	2,54	0,0006
Glutamate receptor, ionotropic, NMDA2B	24410	5,27	0,0001
Calcium-sensing receptor	24247	−1,73	0,0009
Purinergic receptor P2Y	63843	2,67	0,0008
Angiotensin receptor 1a	24180	−1,71	0,0023
Glutamate receptor kainate 3	298521	2,84	0,0010
Glutamate receptor NMDA 2A	24409	3,82	0,0012
Metabotropic glutamate receptor 5	24418	3,10	0,0018
Metabotropic glutamate receptor 3	24416	4,46	0,0034
Glutamate receptor AMPA1 (alpha 1)	691178	2,56	0,0037
Rho-GTPase-activating protein 26	307459	3,41	0,0009
RAB27B, member RAS oncogene family	84590	3,08	0,0004
Protein kinase, lysine deficient 1	116477	4,46	0,0012
Plasma membrane calcium ATPase 4	29600	3,46	0,0029
Protein kinase C alpha	24680	3,00	0,0000
Guanylate cyclase 1, soluble, alpha 2	66012	3,90	0,0016
**Transcription**			
Transforming growth factor, beta 1	59086	−1,40	0,0011
Kruppel-like factor 5	84410	1,51	0,0026
Fibroblast growth factor 2	54250	1,80	0,0017
Transcription factor E2F5	116651	−1,84	0,0022
Retinoblastoma binding protein 7	83712	−1,43	0,0003
Neurogenin 1	29410	−1,43	0,0029
Zinc finger protein HIT-10	170955	6,24	0,0013
Tnf receptor-associated factor 6	311245	3,30	0,0003
General transcription factor	83830	3,07	0,0019
Zinc finger protein 297B	311872	3,16	0,0008
Histone acetyltransferase MYST4	688634	2,74	0,0004
Histone deacetylase 9 isoform 4	314040	2,15	0,0231

To determine the predominant function of the genes which were classified into two or more GO categories, we classed each gene according to its molecular function (nuclear receptor, transcription factor, cellular signaling and undefined) and calculated the percentage of each molecular function for each overlapped group. The results of this analysis indicated that most of the genes classified into more than one GO category behave as transcription factors. Actually, 72% of the genes within the overlapped SM category are transcription factors and 28% do not have a defined function. Within the MT overlapped area, 67% are transcription factors, 4.7% are nuclear receptors, 2.3% participate in cell signaling and 25% have no defined function. In the overlapped area corresponding to SMT, 26% of the genes are transcription factors, 33% nuclear receptors, 19% participate in cell signaling and 22% have undefined function.

### Genes regulating metabolic process

Most of the genes whose expression levels were permanently altered by perinatal protein restriction are involved in the regulation of metabolic processes as determined by the analysis of the microarray data by gene ontology. Indeed, 334 genes out of a total of 997 belong to this functional category. However, many of these genes exhibit one or two additional functions such that only 186 genes are exclusively involved in the regulation of metabolism (Figure 1A). Strikingly, the expression of almost all these genes was down regulated in LP rats in relation to controls. The sub-categorization of this functional cluster indicated an over representation of genes involved in the regulation of protein biosynthesis like the 60S ribosomal proteins L13 and L15, the eukaryotic translation initiation factor 3, the seryl-aminoacyl-tRNA synthetase 1 and the asparatyl-tRNA synthetase 2 (Table 3). The metabolism of glucose and carbohydrates seems also to be hampered by early protein restriction as indicated by the lower expression levels of several genes involved in this metabolic pathway including lactate dehydrogenase C, UDP-glucose pyrophosphorylase, mannosidase alpha class 2A, malate dehydrogenase 2 and xylosyltransferase1. The expression of numerous genes with oxido-reductase activities such as cytochrome P450 family 3 and 20, peroxi-redoxin 3, cytochrome b-5, thioredoxin, NADH dehydrogenase Fe-S and cytochrome oxidase subunit IV was also reduced by perinatal protein restriction (Table 3). This was also the case for some genes regulating cell proliferation like the transcription factor E2F5, the cell growth regulator with ring finger domain 1, cyclin H and the cell division cycle 5-like and cell division cycle 26 genes.

### Cell signaling pathways involved in energy homeostasis and nutrient sensing

The second gene ontology category most affected by perinatal undernutrition regroups genes involved in signal transduction. This category includes the galanin receptor 1 (GALR1) the cannabinoid receptor 1 (CB1) and the glutamate receptor NMDA NR2 (Table 3). All these receptors, whose expression levels were increased more than two fold in LP rats in relation to controls, have been involved in the regulation of food intake and energy homeostasis [24], [25], [26]. To our surprise we found no changes in gene expression of the classical orexigenic and anorexigenic neuropeptides, NPY, AgRP, POMC and CART but this was due to the stringent criteria we used to limit false discovery rate (P<0.003). Indeed, in agreement with our previous observations [15], a close inspection of the microarray data revealed a down regulation of AgRP and POMC with a P-value of, respectively, 0.04 and 0.02. NPY and CART were also down regulated although in this latter case the differences in the levels of gene expression between control and LP rats were not statistically significant. The expression of other neurotransmitter receptors which have not, until now, been directly associated with the regulation of metabolism was also altered by protein restriction. These include several glutamate receptor subtypes and different members of the olfactory receptor family as well as the purinergic receptor P2Y4, the calcium sensor receptor and the angiotensin receptor 1a (Table 3). In addition to altered changes in the expression of membrane receptors, LP rats exhibited up-regulated levels of mRNA encoding enzymes involved in intracellular signaling including several members of the adenylate and guanylate cyclases, Rho and Ras GTPases and protein kinases.

However, the most outstanding result revealed by the analysis of the genes clustered in the signal transduction gene ontology category, was the finding that perinatal protein restriction increased the hypothalamic expression levels of several genes involved in the signal transduction pathway of insulin (Table 4). These genes include insulin receptor substrate-1 (IRS1), phosphatidylinositol-3-kinase (PI3K), phosphoinositide-dependent protein kinase-1 (PDK1), serine threonine kinase Akt3, glycogen synthase kinase 3 beta (GSK3β) and the signal transduction protein CBL. This observation is in line with the results of previous studies which have shown an up-regulation of the PI3K signaling pathway in skeletal muscle of young men with low birth weight [27] or in skeletal muscle of rats submitted to metabolic programming [28]. The constitutive activation of the PI3K pathway in POMC neurons results in hyperphagia, diet-sensitive obesity and leptin resistance [29]. On the other hand, enhanced expression and activity of GSK3β has been reported in the skeletal muscle of diabetic patients and in the muscle of animal models of type 2 diabetes [30], [31]. Therefore, the herein presented results point out the interesting possibility that the increased food intake and metabolic disturbances usually observed in metabolic programmed animals are bring about, at least in part, by disrupted insulin signaling in the hypothalamus.

**Table 4:** Functional and transcriptional gene clusters whose expression in the hypothalamus is coordinately regulated by perinatal protein restriction.

Gene name	Gene symbol	Gene_ID	Fold change	P-value	GO Category
**Enegy homeostasis and nutrient sensing**					
Insulin receptor substrate 1	IRS1	25467	2,21	0,0031	S
Phosphatidylinositol 3-kinase, regulatory subunit, polypeptide 1	PI3K	25513	3,05	0,0017	M, S
3-phosphoinositide dependent protein kinase-1	PDK1	81745	3,25	0,0003	S
Murine thymoma viral oncogene homolog 3	Akt3	29414	1,42	0,0043	S
Glycogen synthase kinase 3 beta	GSK3β	84027	4,04	0,0020	M, S, T
Tuberous sclerosis 1	Tsc1	60445	2,31	0,0026	M, S
Tuberous sclerosis 2	Tsc2	24855	−1,33	0,0029	S
Ecotropic retroviral transforming sequence	CBL	500985	4,37	0,0001	S
**Transcriptional regulation of metabolism**					
Glucocorticoid receptor	GR	24413	3,15	0,0011	M, S, T
Thyroid hormone receptor beta	TRβ	24831	2,69	0,0028	M, T
RAR-related orphan receptor alpha	RORα	300807	7,25	0,0002	M, S, T
RAR-related orphan receptor beta	RORβ	309288	2,77	0,0010	M, T
Peroxisome proliferator-activated receptor gamma	PPARγ	25664	−2,35	0,0015	M, S, T
Retinoid X receptor alpha	RXRα	25271	2,10	0,0007	M, S, T
Estrogen receptor 1	ERα	24890	2,83	0,0002	M, S, T
Androgen receptor	AR	24208	3,33	0,0023	M, S, T
Testicular orphan nuclear receptor 4	TR4	50659	4,24	0,0010	M, T
Germ cell nuclear factor	GCNF	362125	2,97	0,0017	M, T
Peroxisome proliferative activated receptor, gamma, coactivator 1 alpha	PGC-1α	83516	2,31	0,0035	M, S, T
Peroxisome proliferator-activated receptor gamma coactivator 1 beta	PGC-1β	291567	2,18	0,0007	M, S, T
**Circadian clock**					
RAR-related orphan receptor alpha	RORα	300807	7,25	0,0002	M, S, T
Retinoid X receptor alpha	RXRα	25271	2,10	0,0007	M, S, T
Peroxisome proliferative activated receptor, gamma, coactivator 1 alpha	PGC-1α	83516	2,31	0,0035	M, S, T
Circadian Locomotor Output Cycles Kaput	CLOCK	60447	4,61	0,0004	M, S, T

Hierarchical cluster analysis was performed using CLUSTER 3.0 and TREEVIEW software as indicated in the Material and Methods section. The indicated P-value corresponds to the Student's t-test P value. S =  Signal transduction; M =  Metabolism; T =  Transcription.

PI3K and Akt3 are also at the heart of the intracellular transduction cascade initiated by the stimulation by leptin and neurotrophin brain-derived neurotrophic factor (BDNF) of their membrane receptors. Like insulin, leptin and BDNF act in the arcuate and ventromedial nucleus of the hypothalamus to regulate food intake, energy expenditure and peripheral glucose levels by the activation PI3K [32], [33], [34]. Moreover, the PI3K/Akt pathway is an upstream activator of the protein kinase mTOR (mammalian target of rapamycin) which is a critical regulator of nutrient sensing as well as cellular metabolism, growth, and proliferation [35], [36]. Our finding that the expression of PI3K and AKT was significantly increased in the hypothalamus of LP rats in relation to control animals therefore suggests that the fuel sensing mechanisms of the cell are also affected by early protein restriction. In support of this idea, the expression of tuberous sclerosis complex 1 (TSC1), tuberous sclerosis complex 2 (TSC2) and rictor genes was also altered by protein undernutrition (Table 4). The heterodimer TSC1/TSC2 is an upstream negative regulator of mTOR and rictor is a key component of the multi-protein complex mTORC2 which inhibits insulin signaling through a feed-back mechanism involving the phosphorylation of Akt and of several serine residues of IRS-1 [37], [38].

By altering the expression of PI3K and Akt, perinatal protein restriction is therefore disrupting a wide range of neural and hormonal signals involved in the regulation of food intake and energy homeostasis making likely that the herein reported changes in gene expression of this molecule play a key role in the metabolic disturbances associated with nutritional programming.

### Transcriptional control of metabolism

The hierarchical organization of the microarray data by Gene Ontology indicated that 112 genes, representing more than 10% of the genes whose expression level was altered by maternal protein restriction, participate in transcription processes. These include genes encoding growth and cell proliferation factors as well as transcription factors that play a key role in the integration of nutrient, metabolic and endocrine signals and some enzymes involved in the regulation of chromatin structure and function. Transforming growth factor beta (TGFβ), Kruppel-like factor 5 (Klf5), fibroblast growth factor 2 (Fgf2), transcription factor E2F5 (EDF-5), retinoblastoma binding protein 7 (RBBP7), neurogenin 1(Neurog1) and like histone deacetytlase 1 and histone acetyltransferase MYST4 are representative genes of these groups (Table 3).

However, the largest class of genes represented in this group is the nuclear receptor (NR) family. NR are ligand-activated transcription factors that act as nutrient sensors and, by responding to both incoming dietary signals and metabolites generated by the diet, play a key role in the metabolic adaptation of the organism [39]. According to the genomic sequence information available to date, mammals encode 48–49 NR family members. In our microarray analysis, the expression of 11 rat homologous NR members was permanently altered by perinatal protein undernutrition (Table 4). Most of these receptors belong to a functional NR network that regulates lipid metabolism and energy homeostasis as defined by Bookoute et al., [40]. These nuclear receptors are: the glucocorticoid receptor (GR), the thyroid hormone receptor beta (TRβ), the RAR-related orphan receptor alpha (RORα), the peroxisome proliferator activated receptor gamma (PPARγ) and the retinoid X receptor alpha (RXRa) to which the transcriptional co-regulators PGC1a and PGC1b, whose expression is also permanently altered in LP rats, can be added. All these genes are involved in the regulation of lipid and carbohydrate metabolism.

Among the other nuclear receptors whose level of expression was enhanced by early protein restriction, two of them, estrogen receptor alpha (ERα) and the androgen receptor (AR), are clustered into a NR network that links nuclear receptor function to reproduction and development [40] but even these receptors have been shown to play an important role in the regulation of food intake and energy expenditure. Thus AR [41] or ERa [42] knockout mice develop several physio-pathological features characteristic of the metabolic syndrome including body weight gain, hyperphagia and increased visceral adipose tissue. This phenotype can be reproduced by the selective inactivation of ERa receptors in the ventromedial nucleus of the hypothalamus [43] underscoring the importance of hypothalamic NR in the control of energy homeostasis at the whole body level and the relevance of our findings.

### Transcriptional regulation of the circadian clock

Another striking result of our microarray analysis was the finding that the levels of expression of several genes which play an important role in the regulation of the circadian clock are permanently altered by perinatal protein restriction. The circadian clock is an endogenous system that controls a wide variety of physiological processes ranging from body temperature, and heart rate to hormone secretion [44]. It constitutes the link between the temporal availability of nutrients and energy metabolism. At the core of the circadian clock is a set of transcription factors that form transcription-translation feedback loops that maintain cycles of gene expression over a 24 h period. The positive limb of the loop is constituted by the transcription factors CLOCK (Circadian Locomotor Output Cycles Kaput) and Bmal1 (Brain and Muscle Arnt-Like protein 1) whose protein products dimerize to induce the transcription of period (Per1-3) and Cryptochrome (Cry1-2) genes as well as that of other genes which contain E-box elements in their promoters including NR and other genes involved in fatty acid and glucose metabolism. A second regulatory loop involves the co-activator of transcription PGC-1α and the nuclear receptors REV-ERBa and RORa. REV-ERBa is a direct target of the CLOCK-Bmal1complex and represses Bmal1 transcription [45] whereas PGC-1a and RORa act synergistically to activate Bmal1 expression [46], [47].

We found that the mRNA hypothalamic levels of PGC1α and RORα as well as those of CLOCK were significantly increased in LP rats in relation to controls (Table 4). This indicates that protein deficiency during the critical period of perinatal development programs the circadian clock. In line with this hypothesis, we have recently documented that the resulting offspring of Sprague-Dawley rats submitted to the same metabolic programming model as the one used in this study, exhibit significant alterations in the circadian expression profile of diverse genes regulating food intake such as NPY and CART as well as of the three master genes of the circadian clock BMAL1, Period1 and CLOCK [48]. These effects persist long time after weaning and are associated with hyperphagia. Given that the CLOCK/Bmal1 complex regulates also the expression of several genes involved in the control of distinct metabolic processes including glucose and lipid biosynthesis [49], and because of the association between the disruption of the circadian clock and metabolic dysfunction [50], our observations strongly suggest that the susceptibility to develop obesity and insulin resistance induced by protein restriction during early life results, at least in part, from the metabolic programming of the circadian clock.

### Confirmation studies

In order to corroborate, on one hand, the results of the micro-array analysis and to demonstrate, on the other hand, that the differences in gene expression between control and LP rats observed in the hypothalamus of 180 days-old animals are not due to unspecific effects of ageing, we evaluated by real-time RT-PCR the expression levels of the genes clustered into cell signaling and the transcriptional regulation of metabolism, the two gene ontology categories most affected by perinatal protein restriction, using cDNA samples from both 180 and 35 days-old rats. Experiments were performed on 35 days-old pups to show that the transcriptional changes at the time of weaning result from the programming effects of the maternal diet and are not the consequence of the ongoing ingestion of the low protein diet. These experiments confirmed the results issued from the genome-wide analysis and showed that the expression of these two gene clusters is altered in the same way in 35 and 180 days-old rats (Table 5).

**Table 5:** Real time PCR quantification of the genes belonging to the functional and transcriptional gene clusters identified by the genome-wide DNA microarray analysis.

	35 Days		180 Days	
Gene	Controls	Low protein	Controls	Low protein
IRS1	1.00±0.05	1.44±0.13*	1.00±0.02	1.39±0.10**
PI3K	1.00±0.02	1.27±0.04**	0.94±0.11	1.33±0.07*
PDK1	1.00±0.06	1.19±0.O4 *	1.00±0.03	1.32±0.05**
Akt3	1.00±0.05	1.20±0.04*	1.00±0.05	1.36±0.10*
GSK3β	0.94±0.08	1.41±0.08*	0.98±0.07	1.32±0.07*
Tsc1	0.97±0.07	1.39±0.12*	1.01±0.11	1.43±0.01*
Tsc2	1.05±0.03	0.99±0.06	1.00±0.05	1.15±0.05
CBL	0.93±0.08	1.36±0.06*	1.00±0.09	1.25±0.03*
GR	1.00±0.057	0.61±0.07*	1.00±0.07	1.39±0.06*
TRβ	1.03±0.10	1.43±0.11*	1.01±0.07	1.42±0.11*
RORα	1.01±0.06	1.24±0.05*	1.00±0.06	1.57±0.06**
RORβ	1.00±0.03	1.21±0.04*	0.91±0.09	1.44±0.13*
PPARγ	1.00±0.07	0.59±0.08*	1.03±0.04	0.71±0.01***
RXRα	0.97±0.03	1.21±0.007*	1.02±0.02	1.20±0.06*
ERα	1.02±0.09	1.53±0.12**	1.00±0.06	1.62±0.14*
AR	1.07±0.03	1.25±0.04*	1.00±0.04	1.30±0.05*
TR4	1.00±0.04	1.31±0.07**	0.95±0.04	1.33±0.13*
GCNF	1.04±0.08	1.35±0.04**	1.01±0.08	1.32±0.07*
PGC-1α	1.00±0.04	1.27±0.04**	0.96±0.04	1.18±0.02**
PGC-1β	1.00±0.02	1.25±0.06**	1.00±0.05	1.42±0.03*

Quantitative RT-PCR experiments were performed in triplicate using non-pooled mRNA samples from 3 to 4 different animals and β-actin as internal control. The relative amount of the different genes normalized to the endogenous expression of β-actin was calculated by the formula 2^-ΔΔC^_T_ where ΔΔ*C*_T_ =  (*C*_Tgene_ - *C*_T_β_-actin_) LP rat - (*C*_Tgene_ - *C*_Tβ-actin_) control rat.

*P<0.05;

**P<0.01;

***P<0.001 compared with control animals of the same age.

### Conclusions

**Figure 2:**
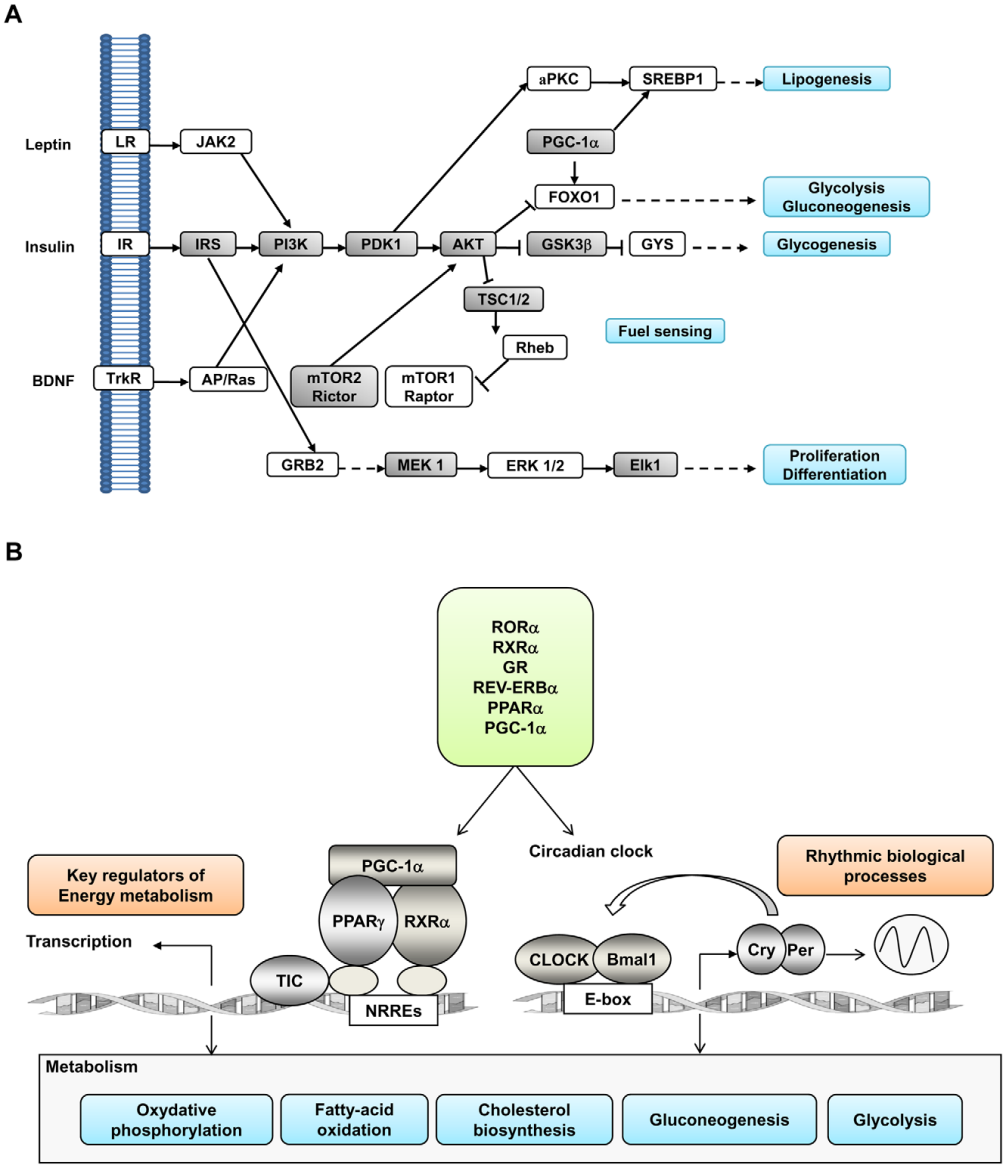
Schematic illustration of the functional (A) and transcriptional (B) gene clusters altered by perinatal protein restriction. (A) Insulin signaling pathway with emphasis on its role in regulating energy homeostasis and nutrient sensing. Lines with end arrows indicate activation, whereas those with perpendicular bars at the end indicate inhibition. Genes exhibiting altered hypothalamic mRNA expression levels in LP rats are represented in shady boxes. (B) Coordinated regulation of energy metabolism and the circadian clock. The diagram illustrates how PGC-1α and nuclear receptors recruit protein complexes and bind, on one hand, to NR response elements (NRREs) within the promoter region of target genes regulating energy metabolism and, on the other hand, to CLOCK and Bmal1, the core genes of the circadian clock. Through the coordinated regulation of the circadian clock and energy metabolism, PGC-1α, RORα and REV-ERBα play an essential role in the adaptation of metabolic gene expression to the temporal availability of nutrients. We found that the expression of the two former genes as well as that of GR, CLOCK and several other NR was permanently altered by perinatal protein restriction. LR  =  Leptin receptor; IR  =  Insulin receptor; TrkR  =  tyrosine kinase receptor.

A number of quantitative or semi-quantitative RT-PCR analyses have been conducted to investigate transcriptional changes in the hypothalamus after nutrient restriction during the critical period of perinatal development. These studies have revealed that protein or calorie restriction during gestation and/or suckling is associated with an altered hypothalamic expression of several anorexigenic and orexigenic genes [15], [18]. Whilst such observations can give a cue about the mechanisms by which undenutrition in early life affects the hypothalamic circuits regulating food intake and energy expenditure, the possibility exists that these transcriptional changes might be a secondary phenomena rather than an initiating mechanism. Here we have performed a comparative genome wide analysis of the transcriptome in the hypothalamus of metabolic programmed rats versus controls. Based on the hypothesis that variations in gene expression levels often reflect changes in gene's activity and that transcriptional networks are at the heart of physiological functions, we identified two gene clusters regulating common cellular processes whose expression is coordinately regulated by perinatal protein restriction. The first one is constituted by several genes of the insulin and leptin signaling pathway which, additionally, act as gate keeper genes for regulation of nutrient sensing (Figure 2). The second cluster encompasses a functional network of nuclear receptors and co-regulators of transcription involved in the detection and use of lipid nutrients as fuel which, in addition, link temporal and nutritional cues to metabolism trough their tight interaction with the circadian clock.

Given the abundance and diversity of cell types constituting the Central Nervous System and the complexity of the interaction between them, one inherent limitation of the microarray and quantitative PCR analysis in brain tissue is their lack of cellular resolution. This remains a challenging issue which is of important concern when we want to determine the functional relevance of transcription changes. Indeed, although many hypothalamic neurons express the same nutrient sensing and signaling transduction proteins, they use the detected information in different, and sometimes opposite, ways to regulate cell activity or gene expression. For instance, AgRP and POMC expressing neurons located within the arcuate nucleus decrease or increase, respectively, their firing rate in response to glucose [51]. Similarly, leptin activates PI3K in POMC neurons but reduces PI3K activity in AgRP neurons [52]. Furthermore, the specific deletion of AMPK from POMC neurons leads to an increased expression ratio of orexigenic versus anorexigenic genes [53]. In contrast, no changes in gene expression are observed after the targeted loss-of-function mutation of AMPK in AgRP neurons [53]. These data have been obtained using a combination of molecular genetics and electrophysiological approaches which allow cell-specific genetic deletion and single cell recordings of activity. However, genome wide expression profiling of individual neuronal cells remains a major technical challenge. Nevertheless, in spite of its lack of cellular resolution, the analysis of gene expression using whole hypothalamus has provided important cues regarding the hypothalamic control of energy homeostasis [54] and microarray analysis of gene co-expression relationships in brain can open new avenues to investigate molecular mechanisms related to altered brain function [55]. In this context, the herein reported results provide a valuable starting point for the direct and systematic experimental analysis of the role of the hypothalamus in the physiopathological disturbances associated with the programming of the metabolic syndrome by protein restriction in early life.

## Materials and Methods

### Animals

All experiments were performed in accordance with the European Communities Council Directive of 24 November 1986 (86/609/EEC). We used an established model of metabolic programming consisting on the restriction of protein to the dam during gestation and lactation as previously described [14]. In brief, eight virgin female Spraguey Dawley rats weighing 200–250 g were obtained from Charles River (France), and placed under a 12 h light/dark cycle (lights on at 07:00) with food and water *ad libitum* for at least ten days before any experimental manipulation. They were then mated to 3 month old males of the same strain (2 females to 1 male). Following confirmation of mating by the visualisation of spermatozoa in a vaginal smear, pregnant dams were housed individually and fed either a control (20 g protein/100 g) or an isocaloric low-protein (LP) diet (8 g protein/100 g) through pregnancy and lactation. Both diets were purchased from Arie Blok (Woerden, The Netherlands) and their composition is provided in Table S1. At delivery, litter size was adjusted to eight pups per dam. At weaning (21 days), all male pups from the control (C) and the low-protein (LP) groups were fed standard laboratory chow. Animals were killed at 6 months of age under non fasting conditions using a rising concentration of CO_2_ and cervical dislocation. Special care was taken to sacrifice all the animals at the same time of the day, 9–10 o'clock in the morning, to prevent the bias due to circadian variations in gene expression. Trunk blood was collected for the analysis of insulin and the metabolites, glucose, triglycerides, cholesterol and fatty acids. Bilateral fat depots from 3 regions (inguinal, retroperitoneal and epididymal) as well as the mesenteric fat pad, were dissected, weighed and summed to provide a measure of body fat, referred to as abdominal fat in the text.

### Hormone and metabolite determinations

Serum from trunk blood was assayed for insulin using an assay kits from Linco Research Inc. Triglycerides, cholesterol and fatty acids were analyzed by enzymatic methods (Triglycérides enzymatiques PAP 150, BioMérieux; Cholesterol RTU, Biomérieux). Serum glucose concentrations were determined with a blood glucose monitor (Accu-Check®, Roche Diagnostics).

### DNA microarrays

Total RNA from the hypothalamus was extracted using the TRIzol reagent (Invitrogene, Cergy Pontoise, France) and subsequently purified using the mini-column purification kit NucleoSpin® from Macherey-Nagel according to the manufacturer's instructions. The quantity and quality of the purified RNA was evaluated using a NanoDrop ND-1000 spectrophotometer and the Angilent 2100 Bio-analyzer. Four micrograms of each RNA sample were sent to the NimbleGen expression array platform. DscDNA synthesis, DNA end-labeling, hybridization, scanning, and data normalization were performed at NimbleGen, which provided the final data file. A total of 10 Rattus norvegicus 385K Arrays (Nimblegen) were conducted using total RNA samples from five control and five LP rats issued from 3–4 different litters. All data is MIAME compliant and the raw data has been deposited in the MIAME compliant database Gene Expression Omnibus (GEO).

### Real-time quantitative RT-PCR

2 µg of purified RNA extracted from the whole hypothalamus of 35 and 180 days-old animals, was reverse transcribed using Superscript II RNAseH- Reverse-Transcriptase (Invitrogen) in a total volume of 20 µl. The resulting cDNA was diluted 50-fold in DNAse and RNAse-free water. Thereafter, 4.5 µl of each cDNA diluted sample was used as template for PCR amplification using SYBR Green (Qiagen, Courtaboeuf, France) as fluorogenic intercalating dye and β-actin as an internal reference for normalization of target gene mRNA expression. The PCR parameters were: an initial denaturation step of 5 min at 95°C followed by 40 cycles of 30 s at 95°C and 30 s at 60°C. The sequences of primers used for the amplification are presented in Table S2.

### Data analysis

To characterize the differences in the profile of gene expression between control and LP rats, and to classify genes and biological samples hierarchically, we used the Gene Cluster program [56]. To this end, the expression level of each gene was first log transformed and then median-centered (relatively to its median expression across all samples), so that relative variations rather than absolute values were used for interpretation. The clustering method used here was an average linkage with the Pearson correlation coefficient as similarity metric. Results were displayed using the TreeView program [56]. In order to validate the most significant differences, Student's *t*-test for paired samples was performed with the XLStat Pro® statistical software (Addinsoft, Paris, France). We used the P-value with a statistical significance of 0.003 corresponding to a false discovery rate of 10%. In this way, two groups of genes corresponding to up-regulated and down regulated genes whose expression was altered by perinatal undernutrition were identified. The two groups of genes differentially expressed in the hypothalamus of LP rats in relation to control animals, were then analyzed using the Gene Ontology (GO) database and the functional classification tool from the DAVID database (http://david.abcc.ncifcrf.gov/). This latter program assigns genes to functional groups within various ontologies and calculates the statistical probability of a particular functional group being over- or under represented. To identify the genes involved in two or more categorized functions, both the up- and down-regulated genes were pooled in their respective Gene Ontology category and distributed in a Venn diagram. The totality of genes for each group (up- and down-regulated) initially submitted to the DAVID database was taken as 100% to obtain the proportion of over-expressed and under-expressed genes in each functional category.

The experimental results of the RT-PCR analysis are expressed as means ± S.E.M. The relative expression levels of the mRNAs in the different hypothalamic samples were calculated using the comparative ΔC_T_ method [57] and β-actin RNA as housekeeping gene. To ascertain the differences between the groups, data were compared by Student's-t-test. Statistical significance was set at P<0.05.

## Supporting Information

Figure S1 Unsupervised hierarchical clustering of gene expression in the hypothalamus of 6 months-old rats born to dams fed a control or a low-protein diet during gestation and suckling. All the animals were fed standard chow since weaning. Cluster colors represent low (green) and high (red) expression levels of probe sets from 10 independent hybridizations corresponding to five control and five LP rats. Note the homogeneous distribution of the genes into two clearly defined up-regulated and down regulated clusters as well as the consistency of the expression changes induced by the maternal diet.
(6.46 MB TIF)

Table S1 Composition of experimental diets.
(0.03 MB DOC)

Table S2 Sequences of primers used for the real time RT-PCR analysis.
(0.04 MB DOC)
