# Supplementary figures and images for: Nutritional Programming in the Rat Is Linked to Long-Lasting Changes in Nutrient Sensing and Energy Homeostasis in the Hypothalamus
Source: PLoS One. 2010 Oct 21;5(10):e13537. doi: 10.1371/journal.pone.0013537 (PMC2958833; doi:10.1371/journal.pone.0013537)

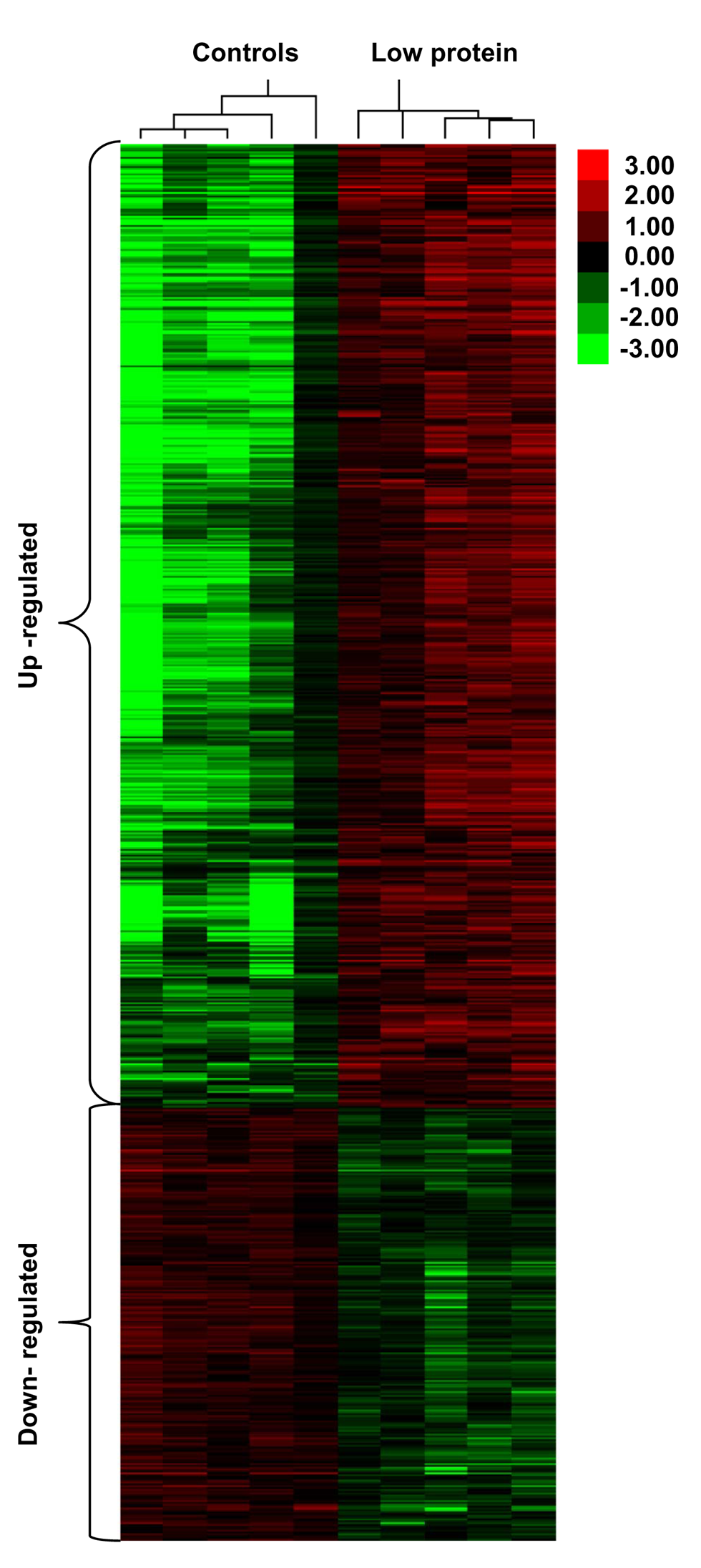

Supplement: Figure S1 — Unsupervised hierarchical clustering of gene expression in the hypothalamus of 6 months-old rats born to dams fed a control or a low-protein diet during gestation and suckling. All the animals were fed standard chow since weaning. Cluster colors represent low (green) and high (red) expression levels of probe sets from 10 independent hybridizations corresponding to five control and five LP rats. Note the homogeneous distribution of the genes into two clearly defined up-regulated and down regulated clusters as well as the consistency of the expression changes induced by the maternal diet. (6.46 MB TIF) [file pone.0013537.s001.tif]
