# Supplementary material for: Nutritional Programming in the Rat Is Linked to Long-Lasting Changes in Nutrient Sensing and Energy Homeostasis in the Hypothalamus
Source: PLoS One. 2010 Oct 21;5(10):e13537. doi: 10.1371/journal.pone.0013537 (PMC2958833; doi:10.1371/journal.pone.0013537)
Supplement: Table S1 — Composition of experimental diets. (0.03 MB DOC) [file pone.0013537.s002.doc]

**Table S1**. Composition of experimental diets (g/kg diet)

| Macronutrient | Control  (20 % protein) | Low protein  (8 % protein) | Standard Chow |
| --- | --- | --- | --- |
| Protein  Casein  Carbohydrate  Cornstarch  Fat  Vegetable oil  Cellulose  Vitamin Mix  Mineral Mix  Energy density (kcal/g)  % macronutrient kcal/g  Protein  Carbohydrate  Fat | 220  631  43  54  10  40  3.8    23  67  10 | 90  761  43  52  10  40  3.8  9.5  80.3  10.2 | 180  630  37  53  10  40  3.6  20.2  70.5  9.3 |

Diet suppliers: Control and low protein diets, AB Diets (Woerden, the Netherlands); standard chow, SAFE, Augy, France.
