# Supplementary material for: Nutritional Programming in the Rat Is Linked to Long-Lasting Changes in Nutrient Sensing and Energy Homeostasis in the Hypothalamus
Source: PLoS One. 2010 Oct 21;5(10):e13537. doi: 10.1371/journal.pone.0013537 (PMC2958833; doi:10.1371/journal.pone.0013537)
Supplement: Table S2 — Sequences of primers used for the real time RT-PCR analysis. (0.04 MB DOC) [file pone.0013537.s003.doc]

**Table S2.** Sequences of primers used for the real time RT-PCR analysis.

| **Gene** | **Forward primer** | **Reversed primer** |
| --- | --- | --- |
| IRS1 | GCCCAGCAACCGCAAAGG | CCAATGTCAGGAGAGCAACTACC |
| PI3K | GGCTTACGCTCCAGTATTTGCTC | GCTGTCGCTCACTCCACTCC |
| PDK1 | TCAGTCCAGTGTGGTGCTATGTTC | GGTGCTTGGTCGGGCTTCAG |
| Akt3 | AACAGAACGACCAAAGCCAAATAC | AATTCATCCTCTCCTCTTCTTGCC |
| GSK3β | CCACCATCCTTATCCCTCCTCAC | TGTCCACGGTCTCCAGCATTAG |
| Tsc1 | GAAGAGGCTGCTGGTCACAATGG | CACTGCTGCTGCTGCTGCTG |
| Tsc2 | GAGCACAGGCAGCACCAGCAG | TGGGTCAGCAAAGGCACATAGGC |
| CBL | AGATGGTGGAGAAGTGCTGGAAG | GTGCGGAGGTGCTGGTAGG |
| GR | CCATTCTAACCATCCTCATCCAC | CGCCATCGCCAAACTTCC |
| TRβ | ACCCAGACAGCGAGACTCTAACC | GGGCAACCTCCGTGTCATCCAG |
| RORα | GCCGAGGTATCTCAGTCACGAAG | TCTGCCGAGGACAGGAGTAGG |
| RORβ | TGCTCTGTTCTCCTCTGCTGTTC | CTGCCGTGATAGTTGGTATCTTGG |
| PPARγ | GGAAGCCCTTTGGTGACTTTATGG | GCAGCAGGTTGTCTTGGATGTC |
| RXRα | GCTGCTGATTGCCTCCTTCTC | GCTCCGTCTTGTCCATCTGC |
| ERα | GGTGGCTCATCCGCTGCTG | CCGAGGCTTTGGTGTGAAGGG |
| AR | CAGTAGACGAGGCAGCAGCATAC | GGGCGTGTGGATGGGTAGGG |
| TR4 | GGCTGTCAGTTGTGAAGGTTGC | GGTTGCGGTGGTGCTTGTTG |
| GCNF | AATCCTGCTCTCGTCCCTCAC | AAGTCGTTCAATCACCTCCATCC |
| PGC-1α | GCCACTACAGACACCGCACAC | ATTCGTCCCTCTTGAGCCTTTCG |
| PGC-1β | GACAGCAGCAGCAGCAGTG | TGGGTGGGCTCTGGTAGGG |
